# Effects of Creatine Supplementation on Brain Function and Health

**DOI:** 10.3390/nu14050921

**Published:** 2022-02-22

**Authors:** Scott C. Forbes, Dean M. Cordingley, Stephen M. Cornish, Bruno Gualano, Hamilton Roschel, Sergej M. Ostojic, Eric S. Rawson, Brian D. Roy, Konstantinos Prokopidis, Panagiotis Giannos, Darren G. Candow

**Affiliations:** 1 Department of Physical Education Studies, Faculty of Education, Brandon University, Brandon, MB R7A 6A9, Canada; forbess@brandonu.ca; 2 Centre for Aging, University of Manitoba, Winnipeg, MB R3T 2N2, Canada; stephen.cornish@umanitoba.ca; 3 Applied Health Sciences, University of Manitoba, Winnipeg, MB R3T 2N2, Canada; umcordid@myumanitoba.ca; 4 Pan Am Clinic Foundation, 75 Poseidon Bay, Winnipeg, MB R3M 3E4, Canada; 5 Faculty of Kinesiology and Recreation Management, University of Manitoba, Winnipeg, MB R3T 2N2, Canada; 6 Applied Physiology & Nutrition Research Group, Rheumatology Division, School of Physical Education and Sport, Faculdade de Medicina FMUSP, Universidade de Sao Paulo, Sao Paulo 01246-903, Brazil; gualano@usp.br (B.G.); hars@usp.br (H.R.); 7 Department of Nutrition and Public Health, University of Agder, 4604 Kristiansand, Norway; sergej.ostojic@uia.no; 8 Applied Bioenergetics Lab, Faculty of Sport and PE, University of Novi Sad, 21000 Novi Sad, Serbia; 9 Department of Health, Nutrition, and Exercise Science, Messiah University, Mechanicsburg, PA 17055, USA; erawson@messiah.edu; 10 Department of Kinesiology, Brock University, St. Catharines, ON L2S 3A1, Canada; broy@brocku.ca; 11 Department of Musculoskeletal Biology, Institute of Life Course and Medical Sciences, University of Liverpool, Liverpool L73 FAK, UK; prokopidis@liverpool.ac.uk; 12 Department of Life Sciences, Faculty of Natural Sciences, Imperial College London, London SW7 2AZ, UK; panagiotis.giannos19@imperial.ac.uk; 13 Faculty of Kinesiology and Health Studies, University of Regina, Regina, SK S4S 0A2, Canada

**Keywords:** supplementation, mental health, depression, amino acids

## Abstract

While the vast majority of research involving creatine supplementation has focused on skeletal muscle, there is a small body of accumulating research that has focused on creatine and the brain. Preliminary studies indicate that creatine supplementation (and guanidinoacetic acid; GAA) has the ability to increase brain creatine content in humans. Furthermore, creatine has shown some promise for attenuating symptoms of concussion, mild traumatic brain injury and depression but its effect on neurodegenerative diseases appears to be lacking. The purpose of this narrative review is to summarize the current body of research pertaining to creatine supplementation on total creatine and phophorylcreatine (PCr) content, explore GAA as an alternative or adjunct to creatine supplementation on brain creatine uptake, assess the impact of creatine on cognition with a focus on sleep deprivation, discuss the effects of creatine supplementation on a variety of neurological and mental health conditions, and outline recent advances on creatine supplementation as a neuroprotective supplement following traumatic brain injury or concussion.

## 1. Introduction

The brain is a highly energetic complex organ, consuming approximately 20% of total resting energy despite accounting for only about 2% of total body mass [1]. Neurons require a constant supply of adenosine triphosphate (ATP) for several cellular processes, including maintaining ion gradients, neurotransmitter exocytosis, and synaptic functioning [2]. Creatine, a nitrogenous organic compound derived from reactions involving the amino acids arginine, glycine, and methionine, is important for resynthesizing ATP, particularly during times of increased metabolic demand (e.g., sleep deprivation, mental health conditions, or neurological diseases) [3,4,5]. Through the reversible reaction catalyzed by creatine kinase, phophorylcreatine (PCr) combines with adenosine diphosphate (ADP) to resynthesize ATP [6]. PCr functions as a high-energy molecule capable of resynthesizing ATP significantly faster than oxidative phosphorylation and glycolytic processes [6,7].

While the vast majority of creatine is synthesized in the kidneys and liver, creatine can also be endogenously synthesized in the brain [8,9,10]. Furthermore, creatine has some ability to cross the blood–brain barrier (BBB; via microcapillary endothelial cells expressing the creatine transporter SLC6A8) [8] and accumulate in the brain. However, creatine uptake in the brain is typically limited in relation to other tissues such as skeletal muscle, possibly because of low permeability of the BBB to creatine and/or astrocytes lack the expression of SLC6A8 [8]. Therefore, total creatine ingestion may need to be higher or for longer periods of time to produce significant effects in the brain compared to skeletal muscle. Over the past few years, there has been an emergence of research investigating the impact of creatine supplementation on a variety of conditions that may be influenced by impaired or altered brain bioenergetics [5].

The purpose of this narrative review is to summarize the current body of research pertaining to creatine supplementation on total creatine and PCr content, explore guanidinoacetic acid (GAA; a creatine precursor) as an alternative or adjunct to creatine supplementation on brain creatine uptake, assess the impact of creatine on cognition with a focus on sleep deprivation, discuss the effects of creatine supplementation on a variety of neurological and mental health conditions, and outline recent advances on creatine supplementation as a neuroprotective supplement following traumatic brain injury or concussions.

## 2. Creatine and Guanidinoacetic Acid (GAA) Supplementation on Brain Creatine and Phophorylcreatine (PCr)

### 2.1. Creatine Monohdyrate Supplementation

Pioneering work in the 1990s by Harris et al. [9] and Hultman et al. [10] demonstrated increased muscle creatine levels following oral creatine monohydrate supplementation. Since the publication of these studies, it has been repeatedly shown that creatine supplementation increases muscle creatine and PCr levels using both nuclear magnetic resonance (NMR) spectroscopy and muscle biopsies (reviewed in Kreider et al. [11]). It appears that the average increase in muscle creatine from creatine supplementation is about 20% with responses that could be characterized as low, medium, or high (≈40%). Intramuscular creatine levels can be further increased when creatine monohydrate ingestion is combined with exercise [9,12], insulin [13], carbohydrate [14], carbohydrate and protein [15], or lipoic acid [16].

There is a plethora of research which has examined the effects of creatine supplementation on skeletal muscle creatine levels, muscle mass and function (e.g., sports performance, strength, resistance to fatigue, adaptation to exercise training). However, research is very limited regarding the efficacy of creatine supplementation on brain creatine and brain function (e.g., cognitive processing, recovery from brain injury) (reviewed in [5,17,18]).

Dolan et al. [18] recently reviewed the effects of creatine supplementation on brain creatine and several observations can be made from that analysis and the original articles. Across 12 individual studies reported in 11 articles, brain creatine content changed from −0.7% to 14.6%, with the majority of studies (*n* = 10) reporting a 3% to 10% increase [19,20,21,22,23,24,25,26]. However, Wilkinson et al. [27] and Merege-Filho et al. [28] reported no increase in brain creatine from creatine supplementation. Furthermore, there are some reports of no change (−0.7%) [24] or decreased brain PCr (−3.1%) [20] from creatine supplementation. Brain creatine was derived using proton or phosphorous nuclear magnetic resonance spectroscopy (H^1^-NMR or P^31^-NMR, respectively), in the aforementioned studies. Independent of methodological differences across studies, this small body of research indicates that creatine supplementation has the ability to increase brain creatine levels, but the magnitude of change is likely less than what is observed in skeletal muscle. There are several possible explanations for the discrepancy between creatine uptake in the brain and skeletal muscle. Skeletal muscle does not have the ability to synthesize creatine. Although >95% of the body’s creatine is stored in skeletal muscle, creatine is manufactured in other tissues (e.g., liver, pancreas, kidneys), enters systemic circulation and gains entry into skeletal muscle via SLC6A8. Dietary creatine (includes supplementation) gains entry into skeletal muscle through the same process. However, the brain has the ability to synthesize creatine [29,30] and therefore appears to be more resistant to the uptake of creatine. Indeed, the absence of SLC6A8 in astrocytes may limit exogenous brain creatine uptake [31]. It could be that the brain relies primarily on endogenous creatine synthesis until there is some sort of challenge to brain creatine status. These challenges, which could cause a decrease in brain creatine could be acute (e.g., sleep deprivation, intense exercise) or chronic (e.g., aging, traumatic brain injury, depression, Alzheimer’s disease, creatine synthesis enzyme deficiencies). As an example, in the case of children with disorders of creatine synthesis, creatine supplementation results in [32,33,34] both clinical improvement and normalization of brain and body creatine levels.

Overall, it appears that brain creatine content can be increased with creatine supplementation. However, it is difficult to compare individual studies where brain creatine was assessed pre- and post-supplementation because the supplementation protocols are heterogeneous (2 to 20 g/d), the populations are different (e.g., patient vs. healthy), the regions of the brain assessed were dissimilar, and while some labs measure brain PCr using P^31^-NMR other research teams measured total creatine using H^1^-NMR. One factor that must be investigated in the future is the optimal dosage of creatine needed to elicit the largest increase in brain uptake in response to supplementation. Similarly, few data assessing simultaneous changes in creatine in multiple tissues (e.g., muscle and brain) are available. It is unlikely that the addition of nutrients such as carbohydrate or protein, or endocrine factors such as insulin will have any effect on brain creatine uptake. Currently, research indicates that brain creatine increases in response to creatine monohydrate supplementation. This increase is smaller than the skeletal muscle response to a similar supplementation protocol.

### 2.2. GAA Supplementation

Being a direct natural precursor of creatine, GAA (also known as glycocyamine; chemical formula: C_3_H_7_N_3_O_2_) has been used to treat neurological diseases for almost 70 years. In 1952, Henry Borsook from Caltech was arguably the first to investigate the effects of supplemental GAA (co-administered with betaine) in poliomyelitis-related disability [35]. The authors reported beneficial effects of glycocyamine therapy in patients affected by acute anterior poliomyelitis, with the presumed therapeutic mechanism entailing enhanced creatine synthesis in target organs, including the brain and skeletal muscle. Succeeding neurological studies from the 1950s derived equivocal results in terms of GAA therapeutic potential, with some showing no clinical improvement in patients with various neurological dysfunctions (e.g., multiple sclerosis, amyotrophic lateral sclerosis, Parkinson’s disease) [36,37], while others demonstrated favorable effects of GAA on specific surrogate indicators of tissue metabolism in poliomyelitis [38], or mild patient-reported benefits from the treatment in motor-neuron disease [39]. However, these pioneering studies did not assess the effects of GAA on tissue creatine levels nor did they evaluate more brain-specific outcomes following GAA administration.

A pivotal pre-clinical trial about supplemental GAA and brain metabolism originates from Robert Bertolo’s lab, with his group being the first to demonstrate increased cerebral creatine levels after GAA ingestion [40]. The authors reported that 3-month-old Yucatan miniature pigs, who were fed control, GAA- or creatine-supplemented diets for up to 19 days, experienced a modest rise in brain creatine (determined with a brain biopsy), with the magnitude tending to be superior in animals fed with GAA compared to controls and creatine-fed pigs. In addition, hepatic, muscle, and kidney creatine levels were higher with GAA versus creatine supplementation. The above findings are corroborated in a randomized controlled crossover trial with healthy men [41], where 28 days of GAA supplementation (3 g/day) resulted in a significant elevation (up to 16.2%) in brain creatine levels assessed via magnetic resonance (MR) spectroscopy in the middle cerebellar peduncle and paracentral grey matter, with the rise greater compared to creatine. The capacity of GAA to increase creatine levels using MR spectroscopy across the human brain was confirmed in several trials [42,43], with published data suggesting a favorable (and brain location-specific) response rate to short-term GAA loading in human cohorts [44]. Besides amplifying cerebral creatine concentrations, GAA (sole or co-administered with creatine) can positively affect several brain performance outcomes, including specific domains of memory in Yucatan miniature pigs [45], or patient- and clinician-reported indices of everyday performance in women with chronic fatigue syndrome [46], and older men and women [47]. Favorable effects of GAA might be due to its role in the control and provision of cellular energy, including its interaction with cellular transporters for taurine and gamma-aminobutyric acid, previously dismissed as un-targetable carriers by other bioenergetics therapeutics (including creatine) (for a detailed review, see [48]).

Besides its direct role in creatine biosynthesis, GAA might impact brain function via several alternative mechanisms reported in animal studies. Takahashi and co-workers [49] investigated possible neuropharmacological effects of GAA in the mammalian brain, demonstrating its impact on modulating cerebral cortex electrophysiology. In line with this, GAA application appears to induce electrophysiological responses of neurons in primary culture and acute murine brain slices [50]. The research group of Angela Wyss from the Federal University of Rio Grande do Sul extensively investigated intra-cerebral administration of GAA in various milieux and revealed that exogenous GAA could repress the activities of several energy-related pathways and enzymes, including Na^+^/K^+^-ATPase [51], creatine kinase [52], and the respiratory chain [53]. Intra-striatal GAA also inhibits glutamate uptake [54], increases acetylcholinesterase activity [55], and decreases antioxidant defense [56], suggesting additional energy-independent roles of exogenous GAA in animal brains. The above effects might be of little relevance to humans since GAA supplementation likely drives no GAA accumulation in the brain of healthy men [57]. Animal studies typically administered GAA in dosages at least two orders of magnitude higher than those used in human nutritional studies. However, supplemental GAA tends to decrease brain choline and glutamate concentrations in healthy men [42,57]; the consequences of these metabolic perturbations (although marginal) for brain health are currently unknown and require further assessment.

**Figure 1:**
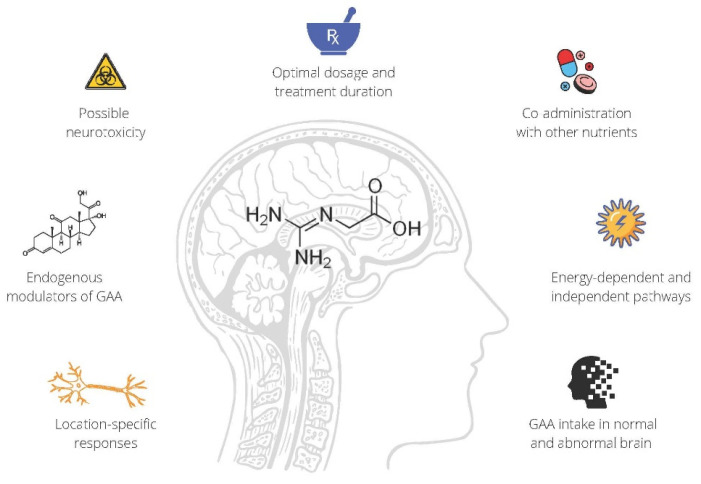
Open questions of guanidinoacetic acid (GAA) supplementation for brain health.

Preliminary data from human studies suggest that GAA can raise brain creatine levels and improve brain performance, with GAA (supplemented alone or along with creatine) perhaps put forward as a promising dietary strategy that could alter biomarkers of tissue bioenergetics in the brain. Nevertheless, more well-designed longitudinal studies are warranted to examine the brain-boosting potential of GAA in various clinical environments, including disorders with neurocognitive impairment and white matter diseases. While future efficacy studies with GAA for brain health are eagerly expected, GAA safety trials remain our utmost priority, considering possible neurotoxicity of exogenous amino acids and derivatives [58]. Addressing both the safety and efficacy of supplemental GAA and other open questions of GAA utilization in nutritional neuroscience (Figure 1) might be the next step forward for the creatine research.

## 3. Creatine and Cognitive Function

Robust evidence that clearly demonstrates the importance of creatine on cognitive function comes from individuals with creatine deficient syndromes, known to deplete brain creatine stores. Creatine deficiency syndrome is characterized by mental and development disorders such as learning delays and seizures [59,60], and importantly these symptoms are reversed, at least in part, by creatine supplementation [32,34,61]. In humans, there are mixed results, with some studies finding some benefits on cognitive functioning [23,62,63,64,65,66,67,68,69,70,71,72], while others found no effect [28,73,74], as recently reviewed by Roschel and colleagues [5]. Similar to cognitive function, research is mixed regarding the effectiveness of creatine supplementation on improving measures of memory. In aging adults (68–85 years) creatine supplementation (20 g/day for 7 days) improved measures of memory (forward number recall, backward and forward spatial recall, and long-term memory) [72]. Rae et al. [75] found improvements in working memory following creatine supplementation (5 g/day for 6 weeks) in vegetarians. In a direct comparison of omnivores and vegetarians, Benton and Donohoe [71] found better memory following creatine supplementation (20 g/day for 5 days) in vegetarians relative to meat eaters. However, others have failed to find beneficial effects from creatine supplementation on measures of memory in children [28], adults [23,65,66,73] and older adults [72,74].

Sleep deprivation is known to impact brain bioenergetics, and it appears that the effects of creatine supplementation in combination with sleep deprivation may enhance cognitive function compared to placebo. However, presently there are only two studies that have investigated cognitive function following sleep deprivation in humans and both were combined with mild to moderate exercise [65,66]. For example, following 24 h of sleep deprivation, creatine supplementation resulted in less change in performance from baseline in random movement generation, choice reaction time, balance and mood state [66]. Furthermore, in a similar experiment by the same group, creatine supplementation attenuated the sleep-deprived loss of complex central executive function [65].

Overall, there is some evidence that creatine supplementation can augment measures of cognitive function. These cognitive effects appear to be more robust when brain bioenergetics are challenged, such as sleep deprivation.

## 4. Creatine for Neurodegenerative Diseases

The relevance of the adenosine triphosphate (ATP)/creatine kinase (CK)/PCr system for central nervous system (CNS) homeostasis is widely recognized. Therefore, increasing brain creatine content is thought to be potentially beneficial for different clinical conditions, such as neurodegenerative diseases [5,76]. Neurodegenerative diseases are commonly characterized as conditions involving a progressive and irreversible loss of neuronal function, thus hampering the ability to perform both cognitive and/or motor tasks. In light of the possible effects of creatine on muscle strength, mass and functionality, its consideration as an adjunct therapy to mitigate disease-related physical impairments is warranted [76,77].

Additionally, oxidative stress, energy depletion and mitochondrial damage are common features in neurodegenerative diseases, to which creatine may act by possibly scavenging reactive oxygen species and increasing energy production [78,79]. This section will highlight the impact of creatine on a variety of neurological diseases.

### 4.1. Amyotrophic Lateral Sclerosis

Amyotrophic lateral sclerosis (ALS) is a neurodegenerative disease characterized by the progressive loss of motor neurons, resulting in muscle atrophy, weakness and paralysis, ultimately leading to death [6]. Using creatine in ALS resides in its potential role as a neuroprotective agent, reducing oxidative stress, attenuating mitochondrial damage and dysfunction and generating energy through ATP resynthesis [78,80].

Despite preliminary evidence in probable/definite ALS patients showing that creatine supplementation resulted in improved physical performance and reduced muscle fatigue [81], other studies in ALS patients with more advanced disease symptoms did not corroborate these findings [82,83,84]. Therefore, the clinical use of creatine in ALS lacks proper empirical support.

### 4.2. Duchenne Muscular Dystrophy

Duchenne muscular dystrophy (DMD) is a life-threatening disease caused by mutations in the dystrophin gene that significantly reduces life span, mainly due to either respiratory or cardiac failure [85].

Creatine supplementation has been shown to improve strength and time to exhaustion in DMD and young Becker’s muscular dystrophy patients [86]. The same authors also found improved bone mineral density among the subgroup of patients able to walk during the trial. These results were further corroborated after a 4-month cross-over trial with creatine supplementation showing improvements in handgrip strength and fat-free mass in DMD patients [87] and a trend towards greater improvement in strength and functionality in DMD [88].

Indeed, results are promising, and more research is warranted to further understand the effects of creatine in DMD and other muscular dystrophies. Importantly, creatine supplementation may confer different effects among the different forms of muscle dystrophies, and results with DMD are not directly generalized to other types of dystrophies.

### 4.3. Huntington’s Disease

Huntington’s disease (HD) is a progressive autosomal dominant neurodegenerative condition characterized by movement, cognition, and behavioral impairments, that can also be fatal. It also encompasses mitochondrial damage and energy metabolism impairment, that can result in increased brain lactate and reduced ATP and PCr regeneration, owing to creatine as a possible promising therapeutic strategy [78,89].

In fact, creatine supplementation has been shown to attenuate disease progression [90]; however, these results are not consistent [91,92]. Two other studies showed that creatine supplementation was able to reduce markers of oxidative injury to DNA, although not accompanied by motor or cognitive improvements [89,93]. More recent evidence [94], with a larger cohort of patients with HD followed for 2 years, showed that a 40 g daily dose of creatine supplementation was unable to positively affect the course of the disease. In summary, results across studies render insufficient scientific support for the use of creatine in this condition.

### 4.4. Multiple Sclerosis

Multiple sclerosis (MS) is characterized as an autoimmune neurodegenerative disease that results in impaired nerve transmission, with symptoms varying across muscle weakness, vision and balance impairments and fatigue. Patients with MS also show alterations in brain and cardiac creatine metabolism, further warranting studies on the effects of creatine supplementation in this disease.

Nonetheless, the few studies performed do not support the use of creatine in this population, as they showed that creatine supplementation did not increase muscle creatine stores [95] nor exercise capacity or muscle power [95,96].

### 4.5. Parkinson’s Disease

Parkinson’s disease (PD) is a one of the most common neurodegenerative diseases, especially in older adults. It is characterized by progressive losses of dopaminergic neurons, resulting in both cognitive and motor impairment, with symptoms ranging from tremor, postural instability, bradykinesia to loss of muscle mass and strength and increased susceptibility to fatigue [97].

Animal studies have shown that creatine supplementation could be potentially neuroprotective by preventing losses of dopaminergic neurons [98]. In humans, creatine has been shown to elicit an improved response to dopaminergic therapy [99] and increased strength and muscle function [100]. Additionally, a 10-g daily dose of creatine has been shown to reduce scores on a disease-specific evaluation scale (Unified Parkinson’s Disease Rating Scale, UPDRS) after 1 year of intervention, suggesting that creatine was able to reduce disease progression. The same consort of investigators observed, however, that disease progression was not different between creatine and placebo groups when follow-up was extended and deemed creatine ultimately ineffective to contain disease progression [101].

Further studies are warranted in order to determine whether creatine, in association or not with exercise and/or other drugs and dietary supplements, may be beneficial for patients with PD.

In summary, creatine supplementation appears to have limited, if any, clinical effect on the progression or management of neurodegenerative diseases. An important scientific challenge that remains in this research area is to determine the optimal creatine supplementation protocol able to increase (or replenish) brain creatine content in order to better understand the therapeutic role of creatine supplementation in neurodegenerative diseases.

## 5. Creatine and Mental Health

The prevalence of mental health disorders is well documented through epidemiological and survey data. An estimated 26–30% of the United States population is affected by a mental health disorder annually [102], while recent data using the 2012 Canadian Community Health Survey suggests that the overall prevalence of mental health disorders in those over 15 years of age was 9.59% [103]. The high prevalence of these disorders and the relatively low adherence rates [104], and limited treatment success with medications for some conditions [105,106], contributes to a substantial economic impact on society [107]. The two most prevalent mental health disorders are depression and generalized anxiety disorder [103].

The critical role of creatine in the brain is well documented through creatine deficiency syndromes, which are characterized by intellectual disability, language delay, seizure disorders, autism spectrum disorder and various movement disorders, with the primary treatment being creatine monohydrate supplementation in an attempt to increase creatine content in the brain [108]. Many mental health disorders have also been characterized to have abnormalities in brain bioenergetics, with some of the more prevalent disorders, such as depression, being associated with low creatine levels in certain regions of the brain [109]. Based on such observations, there has been growing interest in the possible use of creatine monohydrate in various brain/neurological disorders, including mental/psychiatric disorders. The potential therapeutic use of creatine, possible mechanisms of action, and hypothesized clinical implications for psychiatric disorders has been extensively reviewed previously [110].

### 5.1. Depression

Population-based research has established a link between dietary intake of creatine and depression risk in adults [111]. The authors used the National Health and Nutrition Examination Survey to demonstrate a significant negative relationship between dietary creatine and depression. Direct interventional studies using ^1^H-magnetic resonance spectroscopy (^1^H-MRS) have also demonstrated that lower creatine levels in the prefrontal cortex are associated with low mood/increased depression [109]. Even prior to these observations, many different groups have undertaken trials of creatine supplementation in both animals and humans alone and/or in combination with other pharmaceutical interventions to treat depression.

Animal research suggests there may be a sex-dependent relationship regarding creatine and depression [110,112,113,114]. Dietary creatine appears to be more efficacious in female rats compared to male rats [110,112]; however, creatine in combination with other antidepressant drugs does appear to have some benefit in male rodents [114]. The use of creatine has also been investigated in other clinical models where depression is often observed as a secondary consequence of disease, or it results from the treatment. For example, in a mouse model of epilepsy, dietary creatine treatment not only attenuated seizure severity, but it also reduced depressive-like behaviors [115]. Creatine has also been shown to have anti-depressant effects in amyloid β_1-40_ treated mice, a model of Alzheimer’s disease related depression [116]. Chronic corticosterone treatment has also been associated with morphological and behavioral effects that lead to depression [117]. One study has shown that a single dose of creatine can produce morphological alterations that contributed to the improvement of depressive-like behaviors triggered by chronic corticosterone administration, like what was observed with the commonly used antidepressant medication, fluoxetine [118]. The effect of creatine on depression in animal models appears to possibly be related to activation of the rapid activation of the mammalian target of rapamycin complex 1 (mTORC1) pathway, which can occur with a single dose of creatine [119]. Based on the pre-clinical animal model literature, there is clear support for the possible use of creatine in the treatment of depression.

In addition to the larger associative population study mentioned earlier [111], a number of case studies [120] and smaller-scale clinical trials have been published investigating the possible efficacy of creatine supplementation for treating symptoms of depression [21,22,121,122,123,124]. In addition, a recent extensive review focused on the use of creatine for the treatment of depression [125]. Many of the studies have included mostly female participants [21,22,121,122,123,124,126]. Furthermore, most studies have looked at the augmentative effect of creatine to traditional pharmacological interventions [21,121]. Most studies have observed clinically relevant improvements and suggest further investigation into the use of creatine as an intervention for different forms of depression [21,22,120,121,122,123,124]. However, others have not observed any benefit [126].

Collectively, when looking at both the preclinical research and the limited number of small-scale human trials, the research suggests a possible role for creatine supplementation in the treatment of different forms of depression. However, more larger-scale randomized control trials are warranted, and they should include measurements of brain creatine and dietary measures to better understand habitual dietary intake of creatine on the response to such an intervention.

### 5.2. Anxiety and Post-Traumatic Stress Disorder

Generalized anxiety disorder (GAD) is the second most common mental health disorder in Canada, with a reported prevalence of 2.57% [103], while an estimated 70% of the population has experienced a traumatic event in their lifetime and 33% will experience three or more such events [127], which could lead to post-traumatic stress disorder (PTSD). There has been limited investigation into a possible role of creatine in GAD and PTSD. One study has suggested that creatine levels are lower in white matter of patients with GAD that was related to early trauma [128]. Similarly for PTSD, two studies have described reduced creatine levels in the hippocampal region of the brain [129,130]. Despite these observations, there has been little investigation into the possible use of creatine supplementation in these patient populations.

One reported case study involving a 52-year-old woman who was diagnosed with PTSD, depression and fibromyalgia, observed improvements with 4 weeks of creatine monohydrate supplementation [120]. The same group also reported improvements with 4 weeks of creatine monohydrate supplementation in male and female patients diagnosed with PTSD who were receiving psychotropic treatment [131]. This group was deemed resistant to the psychotropic treatment, and with the creatine monohydrate modest improvements in sleep and depression and PTSD symptomology were observed [131]. Based on these limited observations, further work is warranted in the possible use of creatine in the treatment of GAD and PTSD.

## 6. Creatine for Concussion and Traumatic Brain Injury (TBI)

Although the current body of research is limited, the utilization of creatine in the protection and management of concussion and mild traumatic brain injury (mTBI) has been noted as a particular area of interest [5,11,18,132,133,134,135,136]. The current treatment options to address physiological dysfunction following concussion and mTBI is limited to aerobic exercise treatment; however, creatine is postulated to be another option which could address aspects of the neurometabolic cascade associated with a concussion or mTBI [137,138]. Specifically, immediately following a concussion or mTBI a state of hypermetabolism occurs which is then followed by a state of hypometabolism [138,139], however due to limited cerebral energy availability and injury-induced cerebral blood flow anomalies [140], energy supply and demand are uncoupled [137]. Following mTBI, brain creatine content decreases [141,142] and, therefore, creatine supplementation could be beneficial in this scenario.

Currently, in vivo clinical research evaluating the efficacy of creatine supplementation in humans is limited to a pilot study (*n* = 39) that reported beneficial effects in children and adolescents (1–18 years of age) with a severe TBI (Glasgow Coma Scale 3–9 on hospital admission) [143,144,145]. The authors utilized an open label randomized design and found that creatine supplementation (0.4 g/kg/day in an oral suspension administered by nasogastric tube or spoon) was associated with decreased duration of post-traumatic amnesia, intubation and hospital stay, and elicited improvements in neurophysical, cognitive, personality/behavior and social aspects within 3 months and, additionally, improved self-care at 6 months compared to control [143]. In follow-up publications from the same cohort of patients, it was reported that the creatine supplementation resulted in improvements in post-traumatic headaches, dizziness and fatigue [144], as well as dysarthria and lingual problems of understanding [145]. Although clinical research is limited, there are some pre-clinical studies evaluating the effectiveness of creatine supplementation in TBI management.

Creatine supplementation could play a protective role when consumed prophylactically. Sullivan et al. [146] found that mice injected with creatine (3 mg/g/day) for 3 or 5 days prior to a moderate controlled cortical contusion had a 21% and 36% reduction in cortical damage, respectively, compared to placebo at 7 days following injury. The authors also found that rats fed a creatine-enriched diet (1% creatine) for 1 month following a TBI had a 50% reduction in cortical damage. It is suggested that these observed benefits could be through the maintenance of mitochondrial membrane potential, decreases in intramitochondrial reactive oxygen species and calcium, and maintained ATP concentrations [146]. Supporting these findings, Scheff and Dhillon [147] found that rats who received a diet enriched with 0.5% or 1% creatine for 2 weeks prior to a moderate controlled cortical contusion had tissue sparing in the ipsilateral hemisphere compared to a placebo [147]. It was postulated that the observed neuroprotection was due to the inhibition of lactic acid and free fatty acid accumulation found in the creatine supplemented group [147]. Concussion and TBI are associated with the indiscriminate release of the excitatory amino acid glutamate which over activates the N-methyl-D-asparate (NMDA) receptor resulting in increased cellular calcium (Ca^2+^) which causes neuronal death, damage and dysfunction [148]. In a rodent hippocampal embryo cell culture model (>99% neuronal, <1% glial), the presence of creatine (5 mM) did not directly act as an antioxidant, however, creatine did mitigate excitotoxicity induced by a glutamate challenge, increased cellular ATP/PCr concentrations, reduced oxidative stress induced glutamate overflow to the extracellular space, and reduced the Ca^2+^ response to NMDA receptor stimulation [149]. Some evidence exists demonstrating that creatine could be beneficial when supplemented immediately following TBI as well [150]. Saraiva et al. [150] found that creatine supplementation in rats (300 mg/kg/day via intragastric gavage) protected against oxidative stress damage as measured by protein carbonylation and thiobarbituric acid reactive species at 4 and 8 days post-TBI (induced by fluid percussion) but did not provide protection from seizures compared to placebo [150].

Although the current evidence is limited, the utilization of creatine supplementation for the management and protection of concussion and TBI appears promising. The safety of creatine supplementation in humans is well established so future research examining its use in human clinical trials would be of value. Further exploration of creatine supplementation, both prior to and following TBI, is required to determine an optimal consumption protocol.

## 7. Conclusions and Future Directions

It is well established that creatine supplementation can have favorable effects on measures of skeletal muscle mass and performance (i.e., strength). Beyond muscle, accumulating research shows that creatine supplementation and GAA can increase brain creatine content which may help explain some of the preliminary benefits from creatine supplementation on indices of cognition, depression, concussion, and TBI. Research is lacking or inconsistent regarding the efficacy of creatine for treating symptoms of neurodegenerative diseases, anxiety, or PTSD. Future research is needed to determine the mechanistic and clinical effects of longer-term creatine supplementation dosing strategies on brain function and health. Future multifactorial interventions may also be required where creatine is combined with other strategies to enhance cognition or treat neurodegenerative diseases.
